# Adoptive Paternal Age and Risk of Psychosis in Adoptees: A Register Based Cohort Study

**DOI:** 10.1371/journal.pone.0047334

**Published:** 2012-10-10

**Authors:** Mats Ek, Susanne Wicks, Cecilia Magnusson, Christina Dalman

**Affiliations:** Karolinska Institutet, Stockholm, Sweden; University of Queensland, Australia

## Abstract

The association between advancing paternal age and increased risk of schizophrenia in the off-spring is well established. The underlying mechanisms are unknown. In order to investigate whether the psychosocial environment associated with growing up with an aged father explains the increased risk we conducted a study of all adoptive children in Sweden from 1955–1985 (n = 31 188). Their risk of developing schizophrenia or non-affective psychosis in relation to advancing age of their adoptive fathers’ was examined. We found no association between risk of psychoses and advancing adoptive paternal age. There was no support of psychosocial environmental factors explaining the “paternal age effect”.

## Introduction

The association between advancing paternal age and risk of non-affective psychosis, including schizophrenia, in the offspring is well established [1]–[7]. It has been suggested that this relationship is mediated by genetic factors, either through de novo mutations [1] (an increased risk of mutations with increasing number of germ cell divisions) or through genetic traits [7], [8] (parental personality traits which correlate to both genetic vulnerability for schizophrenia and to late fatherhood). A third suggestion is that epigenetic factors [9] play a role, for example by paternal extended exposure to toxins. However, any role of psychosocial environment associated with growing up with an aged father such as increased occurrence of adverse life events e.g. paternal death and illness [10]–[12] has not yet been ruled out [7]. In this study we evaluate the influence of the psychosocial environment by examining whether the risk of developing non-affective psychosis, including schizophrenia, is increased in adopted children who are reared in families with older adoptive fathers compared to those with younger adoptive fathers.

## Methods

### Objectives

The objective of this study is to evaluate if the “paternal age effect” is explained by the psychosocial environment associated with growing up with an aged father. The study design takes advantage of the possibility to differ between the genetic and environmental effect of advanced paternal age in adopted children. A basic assumption is that there is no association between the age of the biological father and the adoptive father.

### Participants

The study population (n = 35 058) was identified via the Multi-Generation Register (held by Statistics Sweden). It consisted of all children adopted by Swedish families and born abroad in 1955–1985 or in Sweden during 1955–1984.

Adoptees from abroad were included if they had immigrated to Sweden before 2 years of age (n = 18 719).

Swedish adoptees were included if they had a known biological mother and two adoptive parents (n = 16 339). Excluded were individuals living with a biological parent at any 5 year point when they were 1–15 years old (n = 1337), not living in a family household (n = 257), or adopted by grandparents or siblings (n = 230).

Individuals from both groups of adoptees were excluded if they emigrated from Sweden or died before 18 years of age (n = 358), had an adoptive father less than 20 years of age (n = 23), or were not living with their adoptive father in the first possible Census (n = 1665).

The final sample consisted of 31 188 adopted children (13 405 born in Sweden and 17 783 born abroad).

The Register of Total Population (held by Statistics Sweden) provided data on emigration and date of death. Household and family data was obtained via linkage to the Swedish Population and Housing Censuses (held by Statistics Sweden).

### Description of Procedures

#### Power

Power calculations showed an 80% chance to detect an increased relative risk for non-affective psychoses with advanced adoptive paternal age of 1.5 with 95% confidence.

#### Schizophrenia and Non-Affective Psychosis

The adopted individuals were followed in the National Patient Register (held by the National Board of Health and Welfare) from 1973 to 2006 regarding in-patient care for schizophrenia (ICD-8: 295 [excluding 295.40, 295.50, 295.70]; ICD-9: 295 [excluding 295E, 295F, 295H] or ICD-10: F20) or any non-affective psychosis including schizophrenia (ICD-8: 295, 297, 298.20–298.99, 299.99; ICD-9: 295, 297, 298C-X or ICD-10: F20-F29).

#### Paternal age

The adoptive father’s age at the adoptees birth, derived from the Multi-Generation Register, was categorized into 4 groups: 20–29 years, 30–34 years, 35–39 years and ≥40 years. The group of fathers aged 30–34 years was chosen as reference group because of low numbers of fathers and few cases in the youngest group (20–29 years). There was information about both biological and adoptive paternal age among 24% of the sample (7588 Swedish-born adoptees).

#### Covariates

We considered the following covariates (described in more detail below): adoptees gender (female v. male) and place of birth (Sweden v. other country), advanced adoptive maternal age (≥35 years old v. <35 years old), adoptive paternal socioeconomic group, residence (urban v. non-urban), and adoptive parents in-patient treatment for psychiatric disorder (yes v. no).

Paternal socioeconomic group was divided into four groups and measured by the first possible census after the child’s birth. The data was derived from the Swedish Population and Housing Censuses where data on occupation was collected every 10 years from 1960 to 1990, with an additional measurement in the census of 1985. The groups were: 1. Non-manual employees including military (1960–1970: code 04, 06, 09; 1980–1990: code 33, 36, 46, 56, 57, 60), 2. Self-employed including upper-level executives (1960–1970: code 01, 03, 05; 1980–1990: code 79, 89), 3. Manual labor (1960–1970: code 02, 07, 08; 1980–1990: code 11, 12, 21, 22), and 4. Occupations that are not identified (1960–1970: code 10; 1980–1990: code 91), students and unemployed non-students (1960–70: code 11, 12; 1980–1990: code 91, 95–99), and people were data on occupation were missing.

Residence was defined by the adoptive father’s place of living in the first possible census after the child’s birth. The data was derived from the Swedish Population and Housing Censuses that was performed every five years from 1960 to 1990. Urban was defined as living in one of the three major Swedish urban areas: Stockholm (code 01 and 02), Malmö (code 12), or Göteborg (code 14). Living in any other parts of Sweden was defined as non-urban.

Finally adoptive parent’s in-patient care for any psychiatric disorder was collected from the National Patient Register. Psychiatric disorder was defined as chapter F in ICD-10 (F0-F99.9) or the corresponding in older versions of the classification (ICD-8: 290–315; ICD-9: 290–319) during the adopted child’s first 18 years of life.

### Ethics

The Research Ethics Committee at Karolinska Institutet, Stockholm, provided ethical approval for the analysis of record-linkage data in the cohort without individual consent (KI Dnr 03-177) in accordance with the Public Access to Information and Secrecy Act and the Personal Data Act. The former govern when data may be released and the latter how data are used. According to the regulations individual consent is not needed when subjects are not participating actively, the information is treated with secrecy, and the results are presented at group level where no individual is possible to identify.

### Statistical Methods

We performed logistic regression analyses using Stata version 9 for Windows. All estimates were adjusted for date of birth since time at risk was different between the oldest and the youngest individuals. The distribution of covariates was examined in relation to exposure and outcome using logistic regression in order to evaluate potentials for confounding. Covariates which were associated with exposure and outcome (either non-affective psychosis or schizophrenia) were added in the final model.

To further clarify the impact of adoptive fathers age analysis was performed on the sub-sample for whom we had information about both biological and adoptive paternal age. A cut-off of 35 years was chosen based on previous literature [13]. The adoptees were grouped into four groups: exposed only to advanced adoptive paternal age, exposed only to advanced biological paternal age, exposed to both and, as reference category, unexposed (neither advance biological nor advanced adoptive paternal age).

## Results

Among the 31 188 adoptees, 371 had been diagnosed with a non-affective psychosis during follow up. Of these, 131 had been diagnosed with schizophrenia. The basic assumption that there was no association between the age of the biological father and the adoptive father was tested on the 24% of the sample (7588 Swedish-born adoptees) for whom we had information about both biological and adoptive paternal age. There was no association (χ^2^ = 13.5, df = 12, p = 0.33).

We did not observe any increased risk of schizophrenia or non-affective psychosis in adopted children in relation to advancing adoptive paternal age (table 1). On the contrary, there was a seemingly lowered risk for non-affective psychosis in adoptees whose adoptive fathers were aged 35–39 years at birth of the child compared to those whose fathers were 30–34 years (OR: 0.7, 95%CI: 0.6–1.0). Gender, place of birth and socioeconomic group correlated with both exposure and outcome and consequently adjustments were performed for these possible confounders one at a time and all together in a final model (table 1). This did not alter the results.

**Table 1:** Odds ratios for schizophrenia and all non-affective psychosis in adopted children in relation to the adoptive paternal age.

	Analytic sample		Schizophrenia						All non-affective psychosis					
Adoptive	n = 31 188		n = 131		Crude^a^		Adj^b^		n = 371		Crude^a^		Adj^b^	
Paternal age	n	%	n	%	OR	95% CI	OR	95% CI	n	%	OR	95% CI	OR	95% CI
**20–29**	4,952	15.9%	19	14.5%	0.9	0.5–1.4	0.9	0.5–1.5	63	17.0%	1.0	0.7–1.3	1.0	0.7–1.3
**30–34**	11,900	38.2%	51	38.9%	1.0	reference	1.0	reference	148	39.9%	1.0	reference	1.0	reference
**35–39**	9,552	30.6%	35	26.7%	0.8	0.5–1.2	0.8	0.5–1.2	92	24.8%	0.7	0.6–1.0	0.7	0.6–1.0
**≥40**	4,782	15.3%	26	19.8%	0.9	0.6–1.5	1.0	0.6–1.6	68	18.3%	1.0	0.7–1.3	1.0	0.7–1.3

a Adjusted for birth date.

b Adjusted for birth date, gender, place of birth and paternal occupational class.

OR = Odds Ratio.

CI = Confidence Interval.

The median paternal age of the biological fathers was much lower compared to that of the adoptive fathers among the 7 588 adoptees with known biological and adoptive fathers (26.0 and 35.2 years respectively). In this group of adoptees, 129 had been diagnosed with non-affective psychosis. As in the whole study population there was no increased risk of non-affective psychosis related to advancing adoptive paternal age compared to unexposed (OR: 1.0, 95%CI: 0.6–1.4). As expected, there was an increased risk related to advancing biological paternal age (OR: 1.4, 95%CI: 0.8–2.5), although not statistically significant. The risk did not increase when advancing adoptive and biological paternal age were combined (OR: 1.3, 95%CI: 0.7–2.3).

## Discussion

This study shows that advancing adoptive paternal age did not increase adopted children’s risk of developing schizophrenia or non-affective psychosis. This was further illustrated in a sub-analysis where we could explore the effect of exposure to advanced biological and adoptive paternal age, separately and in conjunction. As expected, although the power was low in this sub-sample, the estimates support an association with advanced biological paternal age (as found in previous studies) and contradict a relation with advanced adoptive paternal age. Thus, as the first study of this specific issue, we have shown that there is no support of psychosocial environmental factors explaining the so called “paternal age effect”.

### Strengths and Limitations

Our cohort of adoptees was identified from the total Swedish population and the adoptees originated from all parts of the world. The study compared adoptees to each other. The adoptees were placed in families with no regard to the age of their biological father, leaving the genetic risk as a random variable. Altogether, this study design is well suited to explore the role of the psychosocial environment in the association between advancing paternal age and risk of offspring psychosis. The study relies on register-based case ascertainment from the National Patient Register which have been validated and proved reliable for epidemiological studies [14]–[16].

A potential limitation is that families that are adopting may be different from other families. An adopting family needs approval by social services before they are allowed to adopt. This considered, serious life events in the families’ e.g. somatic disorders and deaths are prone to happen in all families. In addition, adjustment for socioeconomic group did not affect the results. However, our measure of socioeconomic group was limited to the father’s occupation. There may be residual confounding by socioeconomic factors. Furthermore, the sample selection, including more affluent families than the general population may affect the external validity.

There is strong support in the literature of an increased risk of schizophrenia in the offspring associated with advancing paternal age [1]–[7]. The underlying mechanism is being debated. The hypothesis that the paternal age effect is due to de novo mutations has been strongly suggested by Malaspina et al [1] and supported by the results of a study showing that advancing paternal age is associated with sporadic rather than non-sporadic cases of schizophrenia [17]. Interestingly, a recent study showed that the father’s age accounts for nearly all the variation of mutations in a child’s genome [18]. Further evidence of the de novo hypothesis is the finding that the risk to develop schizophrenia is decreasing with advancing paternal age in siblings to individuals with schizophrenia [19]. However, the de novo hypothesis has been questioned by Petersen et al [8] who found, in a large sample of more than 2 million individuals, that it was not the father’s age per se, but the father’s age when having his first child that was important. This supports the hypothesis of genetic traits as an explanation of the paternal age effect, i.e. that late childbearing is associated with a genetic disposition for schizophrenia (although not clinically evident). There is also evidence that mothers with schizophrenia have children with aged fathers [7] once again supporting the genetic trait hypothesis. In summary, the underlying mechanisms of the “paternal age effect” are still uncertain, but the evidence supports a combination of trait and de novo mutations rather than psychosocial factors.

This is the first study trying to disentangle nature from nurture and examine whether there are signs of an environmental component in the increased risk of schizophrenia associated with advancing paternal age. We found no association between risk of psychoses and advancing adoptive paternal age. Thus, there was no support of psychosocial environmental factors explaining the “paternal age effect” in our study. However, further studies are needed to rule this out.
